# Evaluation of wavelength ranges and tissue depth probed by diffuse reflectance spectroscopy for colorectal cancer detection

**DOI:** 10.1038/s41598-020-79517-2

**Published:** 2021-01-12

**Authors:** Marcelo Saito Nogueira, Siddra Maryam, Michael Amissah, Huihui Lu, Noel Lynch, Shane Killeen, Micheal O’Riordain, Stefan Andersson-Engels

**Affiliations:** 1 Tyndall National Institute, Lee Maltings, Dyke Parade, Cork, Ireland; 2 Department of Physics, University College Cork, College Road, Cork, Ireland; 3 Department of Surgery, Mercy University Hospital, Cork, Ireland

**Keywords:** Translational research, Medical research, Physics, Applied physics, Techniques and instrumentation, Imaging and sensing, Optical spectroscopy, Biophotonics, Optics and photonics, Colonoscopy, Gastrointestinal diseases, Gastrointestinal cancer, Colorectal cancer, Cancer, Gastrointestinal cancer, Colorectal cancer

## Abstract

Colorectal cancer (CRC) is the third most common type of cancer worldwide and the second most deadly. Recent research efforts have focused on developing non-invasive techniques for CRC detection. In this study, we evaluated the diagnostic capabilities of diffuse reflectance spectroscopy (DRS) for CRC detection by building 6 classification models based on support vector machines (SVMs). Our dataset consists of 2889 diffuse reflectance spectra collected from freshly excised ex vivo tissues of 47 patients over wavelengths ranging from 350 and 1919 nm with source-detector distances of 630-µm and 2500-µm to probe different depths. Quadratic SVMs were used and performance was evaluated using twofold cross-validation on 10 iterations of randomized training and test sets. We achieved (93.5 ± 2.4)% sensitivity, (94.0 ± 1.7)% specificity AUC by probing the superficial colorectal tissue and (96.1 ± 1.8)% sensitivity, (95.7 ± 0.6)% specificity AUC by sampling deeper tissue layers. To the best of our knowledge, this is the first DRS study to investigate the potential of probing deeper tissue layers using larger SDD probes for CRC detection in the luminal wall. The data analysis showed that using a broader spectrum and longer near-infrared wavelengths can improve the diagnostic accuracy of CRC as well as probing deeper tissue layers.

## Introduction

Colorectal cancer (CRC) is third most common type of cancer worldwide and the second most deadly. CRC comprised 10.2% (1.85 million) of the diagnosed cancer cases and 9.2% (0.88 million) of the cancer related deaths in 2018. In the same year, CRC had the second highest 5-year prevalence (10.9% or 4.79 million of the cases)^1,2^. The number of incident cases is estimated to increase by 63.4% until 2040, whereas the number of deaths is estimated to increase by 71.5% in the same period^3^. The projected increased incidence of CRC and the improvement on patient prognosis generated by early CRC detection^4^ has increased the interest in the development of novel techniques to detect precancerous lesions and lesions early in the polyp-carcinoma sequence^5^.

Although current screening methods reduce the risk of CRC-associated mortality^6^, their effectiveness varies due to issues related to suboptimal screening compliance, limitations of test performance and lack of accessibility^4^. With this in mind, initiatives have been taken to increase the range of diagnostic methods including blood and stool-based tests. The results of these tests show only the potential presence of the cancer and further investigation is required to locate potential tumors. Tumors can be visualized and identified by using minimally-invasive methods such as computed tomographic colonography, double contrast barium enema, and capsule endoscopy^4^. However, colonoscopy with biopsy remains the gold standard diagnostic test for evaluation of colonic disease.

Colonoscopy is predominantly a very safe procedure with a very low risk profile of serious complications but does pose significant inconveniences for patients from logistical perspective of bowel preparation, as the patients need to be accompanied home after sedation. Recent research efforts have focused on developing non-invasive techniques to identify cancerous lesions by comparing the techniques capable of generating tissue classification models closely matching the biopsy results. In particular, optical spectroscopy techniques can provide real-time and cost-effective tissue identification, which can provide more accurate and faster than conventional detection methods such as visualization, palpation, and histology^7–20^. One of these techniques is diffuse reflectance spectroscopy (DRS).

DRS is an optical method capable of tissue identification based on the biochemical composition, oxygenation and microstructure of the tissue. DRS technology is based on delivering light to the tissue and capturing the reflected light that travels inside it. The reflected light contains information about the light scattering (related to the tissue microstructure) and absorption (associated with its biomolecular content). The combination of scattering and absorption (optical properties) as well as the geometry of the light source and detector defines the probed depth into the tissue^21^. Since the optical properties vary with the wavelength of the light source, the wavelength range determines the number of biomolecules that can be investigated using DRS.

Previous studies used DRS to distinguish tumor tissue from healthy surrounding tissues in the oral cavity (head and neck cancer)^7^, breast^8,13^, lung^14^, and liver^15,16^. Recent studies have also investigated the detection of colorectal cancer during surgery^9–12,17–20^. In terms of primary colorectal cancer, DRS and related optical spectroscopic techniques have mainly been used to guide colonoscopy by distinguishing the normal mucosa and malignant tissue inside the colon (luminal side). Several of these studies have demonstrated sensitivity and specificity as high as 100% in a limited number of samples and collected spectra^9–12,17–20^. Other optical spectroscopic techniques applied to CRC detection during colonoscopy include fluorescence spectroscopy (FS), time-resolved fluorescence spectroscopy (TR-FS), Raman spectroscopy (RS), Fourier-transform infrared spectroscopy (FTIR)^22–38^. Numerous fluorescence spectroscopy studies achieved both sensitivity and specificity close to 100% for diagnosis^22^. On the other hand, most of the studies performed require contrast agents and/or ultraviolet illumination. This requirement restricts the optical guidance time to the safe limits of ultraviolet exposure and the time contrast agents remain accumulated in the cancerous lesion. Although the diagnostic abilities of label-free FS, TR-FS, RS and FTIR spectroscopy have been investigated for a limited number of samples, the tissue classification performance has also demonstrated sensitivity and specificity up to 100%^23–28,31–38^. Challenges on the repeatability and reproducibility of the classification performance arise from increasing the data collection and analyzing the factors contributing to higher sensitivities and specificities. Factors for DRS include the measured wavelength range as well as the tissue depth investigated by using specific source-to-detector distances (SDD).

Most of the prior studies using DRS and related optical spectroscopic techniques probed only the superficial colorectal tissue by using fiber optic probes with small SDD or collecting hyperspectral images from wide-illuminated tissue areas^39–50^. In addition, previous optical spectroscopy studies have investigated relatively narrow visible and near-infrared wavelength ranges compared to the wavelength range exploited in this study (from 350 to 1919 nm).

In this study, we developed a multivariate analysis model for colorectal cancer detection in ex vivo specimens for future application in colonoscopy settings. We compared its classification performance with previous DRS, elastic scattering spectroscopy, near-infrared spectroscopy and hyperspectral imaging (HSI) studies of other research groups. This performance was evaluated for the broad wavelength range from 350 to 1919 nm as well as ranges used in other studies^39–50^. Furthermore, we investigate the usefulness of the probed tissue depth on cancer identification by using fiber optic probes with SDDs of 630 µm (small SDD for superficial tissue measurements) and 2500 µm (large SDD). Our DRS spectra dataset contained 2889 spectra of freshly excised ex vivo tissues of 47 patients. To the best of our knowledge, this is the first DRS study to investigate the potential of probing deeper tissue layers using larger SDD probes for CRC detection during colonoscopy.

## Methodology

### Clinical study protocol and research ethics

**Figure 1:**
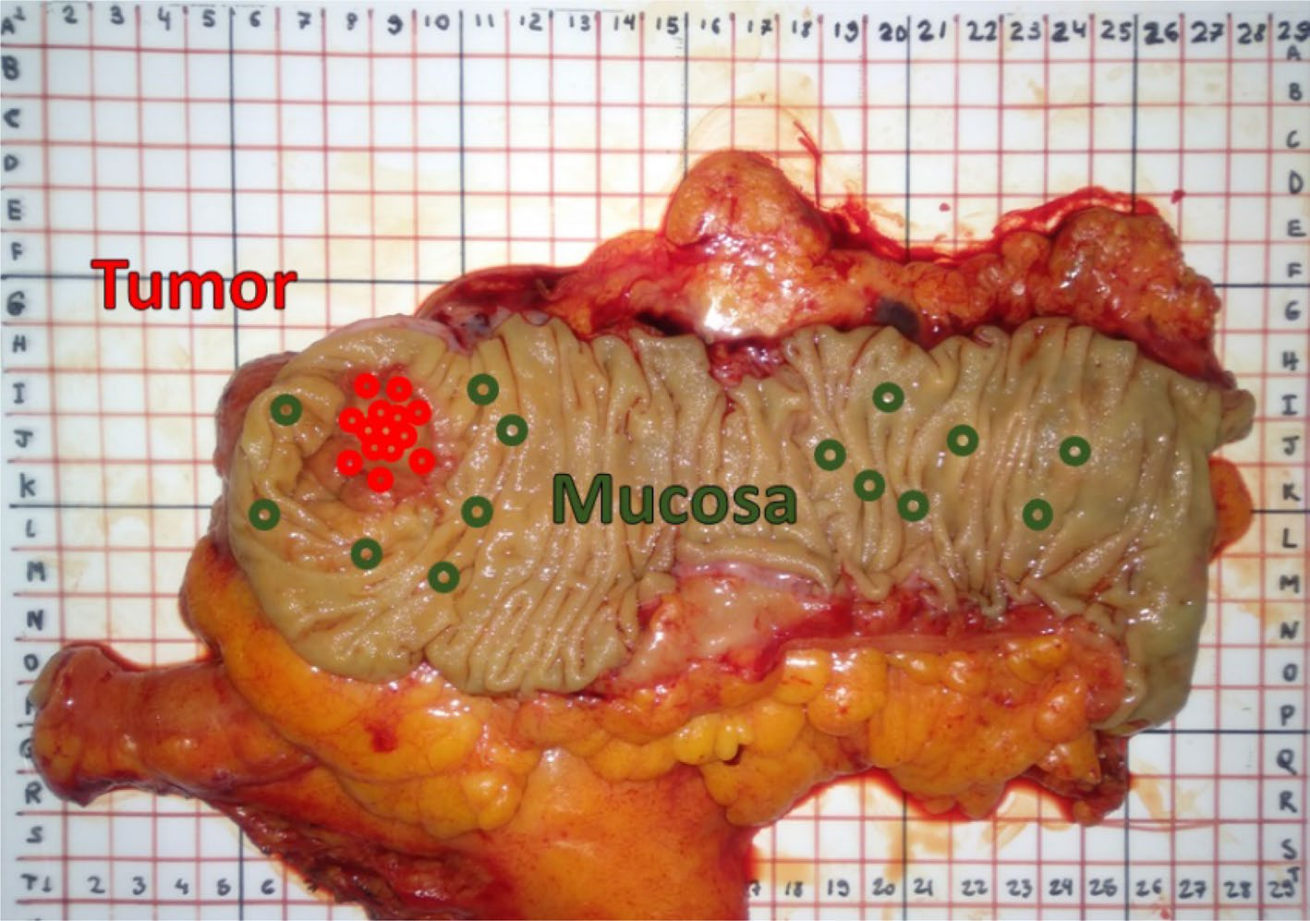
Mucosa and tumor measurement locations on the specimen. Measurements were distributed over a large area in order to obtain a robust dataset containing tissue heterogeneity information.

The study included 47 patients undergoing bowel resection for cancer of the colon or rectum at Mercy University Hospital (Cork, Ireland). Patient demographics are shown in Table 1. The study was performed under the approval of the Clinical Research Ethics Committee of University College Cork. All methods were performed in accordance with the relevant guidelines/regulations. Informed consent was obtained from all participants of the study. The specimen was removed 15–25 min after the blood supply to the specimen had been cut off. Once the specimen was removed from the patients, its lumen was exposed and cleaned with slow running water in order to remove any remaining feces and blood in the surface of the specimen. Then, healthy and cancerous tissues were identified by experienced surgeons prior to the data collection. The time between the specimen removal and the start of the data collection was 40 min on average. The pH was not stabilized by saline solution. The data was collected from ex vivo mucosal/submucosal tissues and tumors of the specimen within an average time of 60 min after surgical resection. Test conditions were kept as uniform as possible throughout the data collection by keeping the tissue moisture with a wet wipe. Approximately 15 sites were measured for healthy tissues (mucosa/submucosa) and 15 for cancerous tissues over a typical area of 100 cm^2^ (Fig. 1).

**Table 1:** Patient demographics and tumor characteristics.

Number of patients	47
**Gender**	
Male	32
Female	15
**Age (years)**	
Median	69
Minimum	40
Maximum	89
Interquartile range	13.5
**Cancer types**	
Adenocarcinoma	46
Carcinoma	1
**Tumor stage**	
pT1	5
pT2	7
pT3	26
pT4	9

The location of every measurement was recorded on a photograph taken with a red–green–blue (RGB) camera. After the acquisition of all optical DRS data, the specimen was returned to the Pathology Department for processing and analysis according to standard protocols. The ground truth of every measurement was obtained by histopathology analysis.

### Diffuse reflectance spectroscopy system and probes

**Figure 2:**
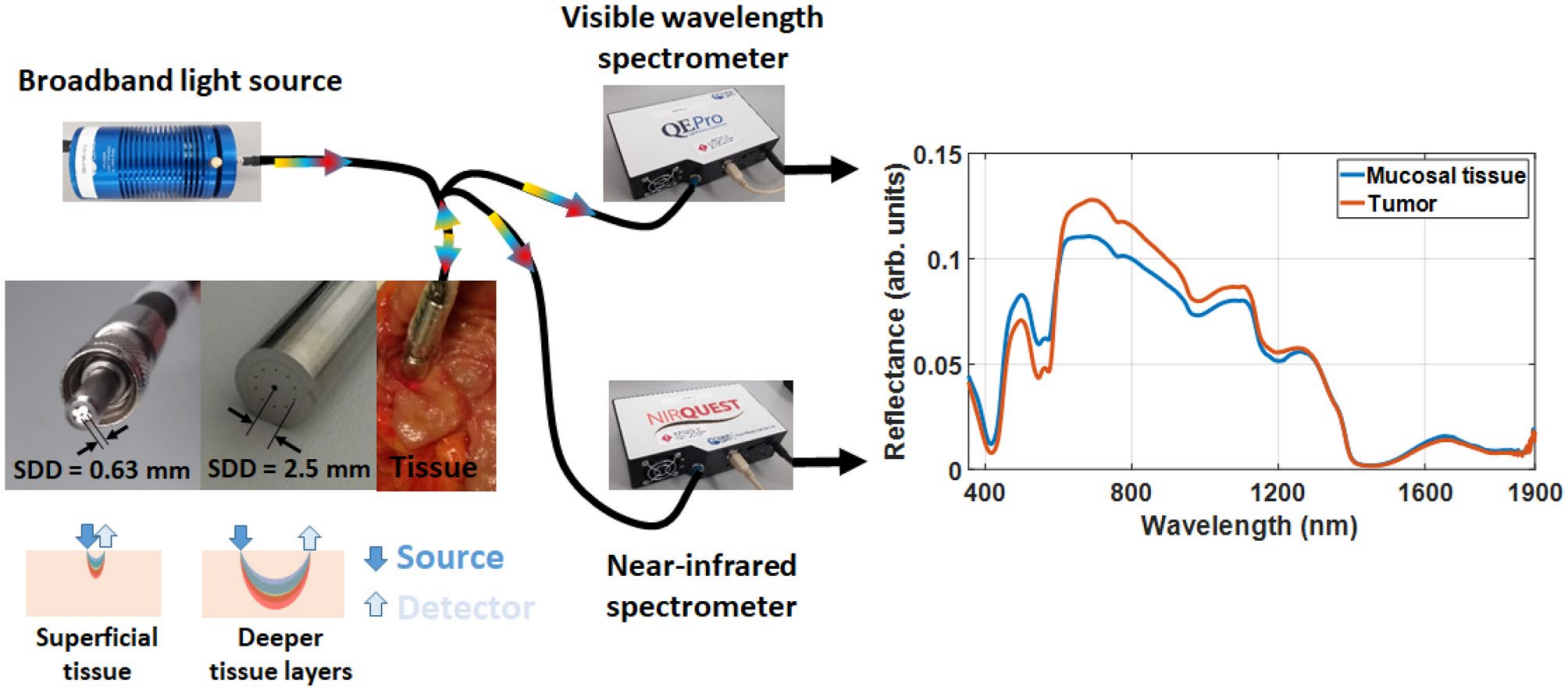
Schematic image of the DRS system. The reflected light can be collected in a wide range of wavelengths and thus, allow the investigation of a larger variety of chromophores in biological tissues.

Our DRS system comprised of a tungsten-halogen broadband light source (HL-2000-HP, Ocean Optics, Edinburgh, United Kingdom) with an emission spectrum ranging from 350 to 2400 nm, which delivers and captures light via optical fibers coupled to both light source and detectors (spectrometers). First, the excitation light is sent through the fiber optic probe. Next, the diffuse-reflected light is collected by optical fibers in the same probe. These fibers deliver the diffuse-reflected light to a visible-wavelength spectrometer (QE-Pro, Ocean Optics, Edinburgh, United Kingdom) and a near-infrared spectrometer (NIR-Quest, Ocean Optics, Edinburgh, United Kingdom). Finally, the light intensity detected by the spectrometers is pre-processed (“Data preprocessing and analysis” section) in order to obtain the tissue reflectance spectra. A schematic drawing of the DRS system is shown in Fig. 2.

In order to obtain the reflectance spectrum preferentially from the superficial tissue (tens of microns deep), we used a quadrifurcated 600-µm-core Low-OH-Silica fiber optic probe (BF46LS01 1-to-4 Fan-Out Bundle, Thorlabs, Munich, Germany) with 630 µm source-to-detector distance (SDD; fiber center-to-center distance represented in Fig. 2). One of the optical fibers was used for illumination, two other fibers were used for collection, and the remaining one was not used during the reflectance measurements. We also used a 2500 µm SDD probe containing one source fiber in the center and 10 collection fibers surrounding it (five for the visible-wavelength detection alternating with five for the near-infrared detection), as seen in Fig. 2. The configuration allows probing deeper tissue layers while achieving higher efficiency on the light collection.

The depth interrogated by each probe was estimated by using a spectral fitting algorithm to extract the optical properties from our DRS measurements. This algorithm was based on forward Monte Carlo simulations of steady-state light transport in multilayered tissues (MCML)^51^. In particular, we used optimized simulations accelerated by graphics processing unit (GPU) reported by Alerstam et al.^52^ by considering tissue as a semi-infinite homogeneous medium with the optical properties (absorption coefficient μ_a_ and scattering coefficient μ_s_) extracted by using a spectral fitting algorithm described in previous studies^12,14,15^. Fluence maps of 5 × 5 mm were generated with simulations using 10 million photon packets, refractive index of the outer medium (η_out_) of 1, tissue refractive index (η_rel_) of 1.4, anisotropy factor (g) of 0.9, and 50 μm of radial and depth resolution. Based on these fluence maps, the photon hitting density maps of 3.75 mm × 5 mm were computed by converting cylindric to cartesian coordinates and multiplying the fluence maps at the positions of the source and collection fibers. Then, the position of the maximum photon hitting density value at the mean position between the source and detector was taken as the average probed depth for each wavelength. Then, the minimum and maximum probed depth were reported in this study in order to show the independence of the datasets acquired with each probe and the importance of evaluating the classification performance at each tissue probed volume.

### Optical data collection protocol

Prior to and after each run of clinical data collection, we collected the background and reference measurements. The reference measurements were taken on a specialized holder able to keep a fixed distance of 2 mm and 6 mm between the small SDD or large SDD probe and the reflectance standard (FWS-99-01c, Avian Technologies LLC, New London, USA), respectively. The fixed distance allows the comparison of the reflectance data across patients. Prior to each set of tissue measurements, the probes were covered with polyvinyl chloride (PVC) film to avoid possible contamination. The same probes were used for every clinical measurement throughout this study. During the tissue data collection, measurements were performed by positioning the probe 90 degrees from the tissue surface. A total of 1363 spectra were collected for the small SDD probe (630 µm SDD) and 1526 for the large SDD probe (2500 µm SDD).

### Data preprocessing and analysis

The data preprocessing comprised of the background subtraction and subsequent division of the captured signal (tissue reflected intensity) by the reflected intensity of the reference (reflectance standard):

$$Reflectance\left( \lambda \right) = \frac{Tissue \;reflected\; intensity - Background\; intensity}{{Reference \;reflected \;intensity - Background \;intensity}}.$$

Once the reflectance spectra were obtained for both visible and near-infrared spectra, these spectra were merged based on the overlapping spectral region between the two spectrometers (from 1095 to 1130 nm). The merging was performed by first doing an interpolation of the overlapping region of the two spectrometers. The interpolated data were used for a weighted sum following:

$$Reflectance\left( \lambda \right) = \mathop \sum \limits_{i = 0}^{100} \frac{{\left( {\left( {100 - i} \right) \times reflectance \;of\; VIS \;spectrometer + i \times reflectance \;of\; NIR \;spectrometer} \right)}}{100}$$

The result is a smooth reflectance curve where the reflectance measured by each spectrometer has more contribution in the wavelength regions where they are most sensitive. In order to prepare the reflectance data for classification, the data was centered and scaled/normalized between − 1 and + 1. This scaling ensures the contribution of data at each wavelength is similar for building the classification models used in this study.

**Figure 3:**
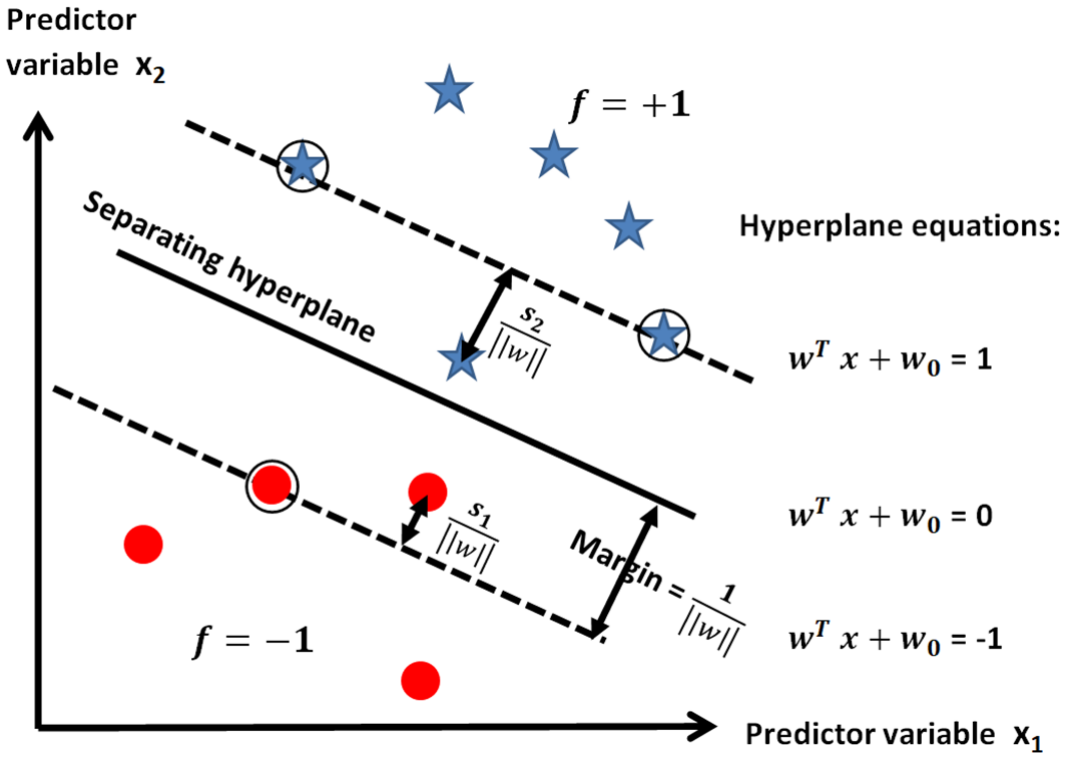
Graphical explanation of SVM hyperplane and accepted margins as defined by parameters involved on the hyperplane equations and slack variables $s_{i}$. The stiff condition of hard margins represented in this schematic is relaxed by slack variables, which act as local quantities that add flexibility to the margin.

Our data analysis consisted of building tissue classification models based on support vector machines (SVMs) and evaluating their accuracy. The training set for the classification was $\left\{ {{\varvec{x}}_{i}^{m} , y_{i} } \right\}_{i = 1}^{n}$ with n samples (e.g. number of reflectance spectra) and with m features (e.g. number of wavelengths). The SVM classifier finds the best hyperplane that separates two types of samples described by the class labels $y_{i} \in \left\{ { - 1, + 1} \right\}$ (e.g. normal mucosa and tumor) based on a predictor variable ${\varvec{x}}_{i}^{m}$ (e.g. reflectance values), where i = 1, 2, …, n. The hyperplane was calculated by:

$$\mathop {\min }\limits_{{w,w_{0} ,s_{i} }} \left( {\frac{1}{2}\left| {\left| {{\varvec{w}}^{2} } \right|} \right| + C_{i} \mathop \sum \limits_{i} s_{i} } \right){\text{subject to }}y_{i} \left( {{\varvec{w}}^{T} {\varvec{x}} + w_{0} } \right) \ge 1 - s_{i}\,\, and \,\, s_{i} \ge 0,$$

where $\user2{w } \in {\varvec{R}}^{{\varvec{p}}}$ and $w_{0}$ are parameters that define the hyperplane, $C_{i}$ is the regularization parameter (or penalty strength) and $s_{i}$ are the slack variables. $s_{i}$ was used to measure the level of error accepted on the hyperplane margin, while $C$ defines the penalty for misclassifications. The two parameters together determine the width of the accepted margin on the hyperplane (Fig. 3).

The classification is given by a sign function which determines the side of the hyperplane a given sample falls into:

$$f\left( {\varvec{x}} \right) = sign\left( {\mathop \sum \limits_{i = 1}^{n} y_{i} C_{i} K\left( {{\varvec{x}},x_{i} } \right) + w_{0} } \right)$$

where the $K\left( {x_{i} ,x_{j} } \right)$ represents the Kernel function:

$$K\left( {x_{i} ,x_{j} } \right) = y_{i} K\left( {{\varvec{x}},x_{i} } \right) = \emptyset_{i} \emptyset_{j} ,$$

where $\emptyset$ is the transformed space for non-linear mapping.

The Kernel function for polynomial kernels is given by:

$$K\left( {x_{i} ,x_{j} } \right) = \left( {x_{i} x_{j} + 1} \right)^{p}$$

where p is the order of the polynomial. The Kernel function for Gaussian kernels is defined by:

$$K\left( {x_{i} ,x_{j} } \right) = \exp \left( { - \frac{{\left( {x_{i} - x_{j} } \right)^{2} }}{{2\sigma^{2} }}} \right).$$

Classification models based on SVMs were built by using the algorithms available in the classification learner app of MATLAB R2016a. All SVM classifiers used the penalty strength $C$ (or Box constraint level in MATLAB) as 1. Linear, quadratic and cubic SVMs used MATLAB automatic heuristic procedure based on subsampling to select an appropriate Kernel scale factor. The software divides all elements of the predictor matrix (scaled reflectance values) by the Kernel scale factor and applies the kernel norm to compute the Gram matrix of the Kernel function. Fine, medium and coarse Gaussian SVMs used Kernel scale factors of 0.5, 2 and 8, respectively.

The models for the broadest wavelength range of this study (from 350 to 1919 nm) were built by several types of SVM algorithms using linear, quadratic, cubic, fine Gaussian, medium Gaussian and coarse Gaussian Kernel functions. In addition, quadratic SVMs were used to develop models on wavelength ranges covered by previous studies reported in the literature. The performance assessment of all generated classification models took into account their sensitivity, specificity, accuracy and area under the receiver operating characteristic curve (AUC). The performance parameters were obtained by using twofold cross-validation for 10 iterations with random sampling. In order to do this, the dataset was first separated into training and tests sets of equal size. Next, the model was generated using the training set. Then, the model was applied to classify and validate the test set. Afterwards the test and training sets were switched and the same process repeated. At the end of this process, the output was the mean of each classification performance parameter. Then, the process is repeated 10 times. Then, the mean and standard deviation of the of the output of the 10 iterations were determined and reported in this study. The reproducibility of these parameters was assessed by the obtained standard deviations. Although fivefold cross-validation is the usual type of validation, this study used twofold cross-validation in order to show the patterns used for tissue differentiation in our dataset can be found by using 50% of the data as training set instead of 80%. Twofold cross-validation also allows the model to be tested in a larger dataset compared to fivefold cross-validation, which means that successful classification will occur only if the model is robust enough to describe half of the dataset by using half for training. In this case, successful classification means stronger potential of generalization of the model upon increase in sample size, especially compared to validation results using more than 50% of the dataset for training and less than 50% for testing.

### Comparison with previous studies

The classification performance for colorectal cancer detection achieved in this study was compared to similar studies investigating DRS, elastic scattering, near-infrared spectroscopy (reflectance modality) and hyperspectral imaging^39–50^. This comparison involved listing the type of tissue evaluation (in vivo or ex vivo), number of patients, wavelength range, source-to-detector distance, types of tissue analyzed, number of analyzed spectra, tissue types used for classification, and diagnostic performance metrics (sensitivity, specificity, accuracy, and AUC). In order to consider comparable probed depths and biomolecules, the performance metrics of this study took into account the classification using similar SDD and wavelength ranges. The classifier used for all the comparisons was the quadratic SVM.

## Results

### Tissue classification

In order to choose which classifier would be used for comparison of the performance achieved with different wavelength ranges, we first evaluated classification models using SVMs and the entire wavelength range of the diffuse reflectance spectra. The performance of SVMs with linear, quadratic, cubic, fine Gaussian, medium Gaussian and coarse Gaussian Kernel functions on the dataset were assessed for the small SDD probe (n_normal tissue_ = 728 and n_tumor_ = 635) and the large SDD probe (n_normal tissue_ = 804 and n_tumor_ = 722). The probed depth for the small probe ranges from 300 to 1000 μm, whereas the one for the large probe ranges from 800 to 2000 μm (data not shown). Then, the tissue volume interrogated by each probe is different and can affect the classification performance parameters. Sensitivity, specificity, accuracy, and AUC are shown in Tables 2 and 3.

**Table 2:** Comparison of the classification performance of SVM classifiers for the dataset of the small SDD probe (n = 1363) containing reflectance values from 350 to 1919 nm.

Type of SVM	Sensitivity (%)	Specificity (%)	Accuracy (%)	AUC
Linear	95.2 ± 1.9	91.8 ± 1.1	93.6 ± 1.5	0.970 ± 0.007
Quadratic	93.5 ± 2.4	94.0 ± 1.7	93.8 ± 2.0	0.971 ± 0.014
Cubic	93.8 ± 0.5	93.3 ± 1.9	93.6 ± 1.2	0.968 ± 0.011
Fine Gaussian	86.4 ± 2.3	90.1 ± 1.3	88.2 ± 2.1	0.960 ± 0.007
Medium Gaussian	90.7 ± 0.3	88.0 ± 1.7	89.4 ± 1.0	0.965 ± 0.005
Coarse Gaussian	80.8 ± 3.7	79.9 ± 2.5	80.4 ± 3.1	0.878 ± 0.023

**Table 3:** Comparison of the classification performance of SVM classifiers for the dataset of the large SDD probe (n = 1526) containing reflectance values from 350 to 1919 nm.

Type of SVM	Sensitivity (%)	Specificity (%)	Accuracy (%)	AUC
Linear	93.1 ± 0.2	91.9 ± 0.6	92.5 ± 0.4	0.963 ± 0.004
Quadratic	96.1 ± 1.8	95.7 ± 0.6	95.9 ± 1.2	0.987 ± 0.005
Cubic	94.9 ± 0.7	95.5 ± 0.4	95.2 ± 0.5	0.983 ± 0.004
Fine Gaussian	82.4 ± 1.9	95.1 ± 1.3	88.4 ± 1.6	0.970 ± 0.005
Medium Gaussian	94.7 ± 0.8	89.6 ± 0.6	92.3 ± 0.7	0.975 ± 0.005
Coarse Gaussian	90.5 ± 1.5	85.1 ± 0.8	87.9 ± 1.2	0.935 ± 0.005

Table 2 shows that the classification performance (small SDD probe) for the polynomial (linear, quadratic and cubic) SVMs is higher than the Gaussian SVMs. The use of the linear SVM leads to higher sensitivity values, whereas quadratic and cubic SVMs can be used to obtain higher performance metrics in exchange for lower sensitivity. The increase in all performance metrics when comparing the coarse Gaussian SVMs with the fine and medium Gaussian SVMs suggests the optimum decision boundary is reasonably narrow. The narrower the boundary is, the higher the specificity and the lower the sensitivity achieved.

Table 3 indicates the classification performance for the large SDD probe has similar trends as that of the small SDD probe in terms of the comparison between polynomial and Gaussian SVMs. On the other hand, the decrease in the performance metrics when decreasing the Kernel scale factors (i.e. using finer Gaussian SVMs) is not as pronounced. In addition, the quadratic SVM model achieved the highest performance metrics. Since the quadratic SVM led to the highest performance for both probes, we used this SVM for comparing the performance metrics with other studies.

The comparison between the best performance metrics of the small and large SDD probes indicated an average gain of 2.6% sensitivity, 1.7% specificity, 2.1% accuracy and 0.16 AUC for tissue classification between 350 and 1919 nm. This suggests probing deeper tissue layers with this wavelength range may improve colorectal cancer detection during colonoscopy.

The performance metrics ((96.1 ± 1.8)% sensitivity, (95.7 ± 0.6)% specificity, (95.9 ± 1.2)% accuracy and 0.987 ± 0.005 AUC) for the large SDD probe are sufficiently high to show potential comparison with other techniques. Furthermore, these metrics are robust as they are based on twofold cross validation of a 1526 spectra dataset. The same robustness is shown for the dataset of the small SDD probe. The classification performance can be further improved by combining DRS with other optical techniques such as fluorescence spectroscopy (FS), time-resolved fluorescence spectroscopy, Raman spectroscopy, Fourier-transform infrared spectroscopy.

### Usefulness of the extended wavelength range

Based on the classification performance on the wavelength ranges of previous studies^39–50^, we analyzed the potential benefit of using the extended wavelength for colorectal cancer (CRC) detection. In order to be consistent with the findings of previous studies, this analysis consisted of the comparison of the sensitivity, specificity, accuracy and AUC using the small SDD probe (Table 4).

**Table 4:** Performance metrics of each wavelength range analyzed using the quadratic SVM classifier on the dataset of the small SDD probe.

Analyzed wavelengths	Sensitivity (%)	Specificity (%)	Accuracy (%)	AUC
From 350 o 760 nm	84.7 ± 2.4	87.0 ± 2.3	85.8 ± 2.4	0.922 ± 0.014
From 350 to 800 nm	87.4 ± 2.1	87.3 ± 2.7	87.4 ± 2.4	0.933 ± 0.009
From 400 to 440 nm and from 540 to 580 nm	84.2 ± 2.9	86.3 ± 2.2	85.2 ± 2.6	0.917 ± 0.012
From 400 to 1000 nm	90.5 ± 1.2	92.0 ± 0.5	91.2 ± 0.9	0.963 ± 0.004
From 405 to 665 nm	85.6 ± 1.4	90.0 ± 0.7	87.7 ± 1.1	0.930 ± 0.010
From 405 to 750 nm	86.8 ± 0.5	89.0 ± 0.3	87.8 ± 0.4	0.927 ± 0.005
From 470 to 700 nm	82.3 ± 2.2	86.0 ± 0.8	84.1 ± 1.6	0.900 ± 0.008
From 415 to 425 nm, from 495 to 505 nm, from 525 to 545 nm, and wavelengths 465 nm and 625 nm	74.5 ± 0.4	83.0 ± 0.8	78.5 ± 0.6	0.863 ± 0.005
405.235 nm, 406.42 nm, 408.794 nm, 414.736 nm, 422.48 nm, 468.76 nm, 559.489 nm, 577.506 nm, 594.342 nm, 957.948 nm, and 1000.31 nm	78.2 ± 0.7	86.0 ± 0.5	81.9 ± 0.6	0.903 ± 0.005
From 900 to 1300 nm	86.9 ± 3.5	88.0 ± 3.7	87.4 ± 3.6	0.921 ± 0.020
From 1000 to 1919 nm	92.1 ± 1.2	92.3 ± 1.4	92.2 ± 1.3	0.950 ± 0.011
From 350 to 1919 nm	93.5 ± 2.4	94.0 ± 1.7	93.8 ± 2.0	0.971 ± 0.014

Table 4 shows the performance metrics for the wavelength ranges covering near-infrared wavelengths are higher than those achieved by analyzing only the visible wavelength range. Moreover, the broader and longer the near-infrared range evaluated, the higher the performance is achieved. The assessment of wavelengths from 1000 to 1919 nm showed similar tissue classification performance compared to the entire wavelength range analyzed in this study (between 350 and 1919 nm). This fact could not be observed by solely analyzing the diagnostic ability of previous studies, as comparison across studies was impossible due to insufficient overlap among investigated wavelength ranges.

## Discussion

### Relevant biochemical and structural differences for CRC detection

**Figure 4:**
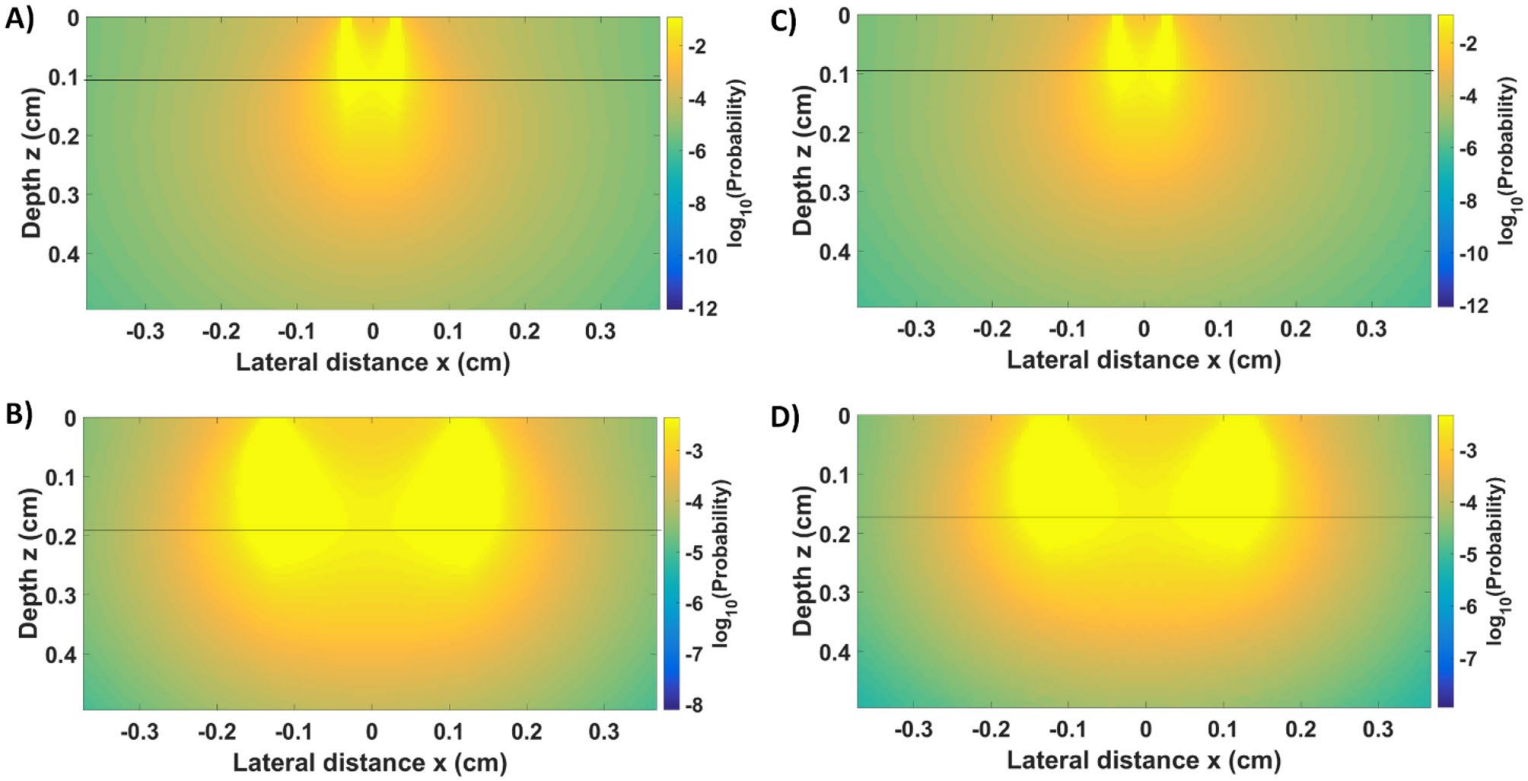
Photon hitting density (PHD) map generated from fluence maps of forward Monte Carlo simulations of light propagation in mucosa (**A**,**B**) tumor (**C**,**D**) tissues at 1150 nm. The black line shows the position of maximum in the middle of the PHD map, which indicates the probed depth at SDD for the (**A**) small probe (630-μm SDD) and (**B**) large probe (2500-μm SDD).

From a clinical perspective, biomolecular changes probed by optical techniques^53–79^ can potentially obviate the need for multiple biopsies or polypectomies of normal mucosa as well as identify sessile serrated polyps, which may be difficult to recognize during colonoscopy at times^7–20^. The potential of optical spectroscopy for colorectal cancer (CRC) detection in ex vivo specimens or in vivo during colonoscopy has been evaluated for superficial tissues (small SDD probes) in several wavelength ranges^39–50^. To the best of our knowledge, this is the first study that uses DRS information from deeper tissue layers (2500 µm SDD) to detect cancerous tissues in the luminal wall of ex vivo specimens for colonoscopy applications. Even though light penetrates deeper for certain wavelengths, if small SDD probes are used, the collected light is predominantly reflected from the tissue surface (Fig. 4). Figure 4 shows that the probed depth in the near-infrared region where the maximum probed depth is achieved for both small and large probes. The probed depth of the small probe typically varies between 0.5 and 1 mm (> 0.8 mm for most wavelengths), while that of the large probe varies mostly between 0.5 and 1.9 mm (> 1.4 mm for most wavelengths; data not shown).

Previous studies used small SDD probes and visible/near-infrared wavelength ranges to investigate signals related to the biochemical composition (related to light absorption) and microstructure (associated to light scattering) of the tissue surface. This biochemical composition includes concentrations of oxyhemoglobin, deoxyhemoglobin, met-hemoglobin, bile, bilirubin, β-carotene, water, lipid, collagen, elastin, and other biomolecules. The tissue microstructure can be investigated due to refractive index mismatches caused by multicellular structures (such as vessels, fibers, etc.), cells size, number of organelles (e.g. mitochondria), composition of tissue layers, extracellular matrix, cell and organelle membranes, collagen fibers and fibrils, and other factors. Once factors such as tissue microstructure, biochemical composition and probed depth are better understood, novel designs of DRS and hyperspectral imaging systems can be proposed to improve the diagnostic accuracy for CRC.

In terms of colonoscopy applications, primary CRC needs to be differentiated from the surrounding normal mucosa (the inner lining of the colon). The intraluminal optical guidance may be affected by other tissue layers apart from the mucosa, especially when using large SDD probes. The colorectal wall is composed of layers of mucosa, submucosa, muscularis propria, and serosa ordered from the intraluminal to the extraluminal side^49^. Each tissue layer differs in their microstructure and biomolecular content which changes the scattering and absorption properties, respectively.

**Figure 5:**
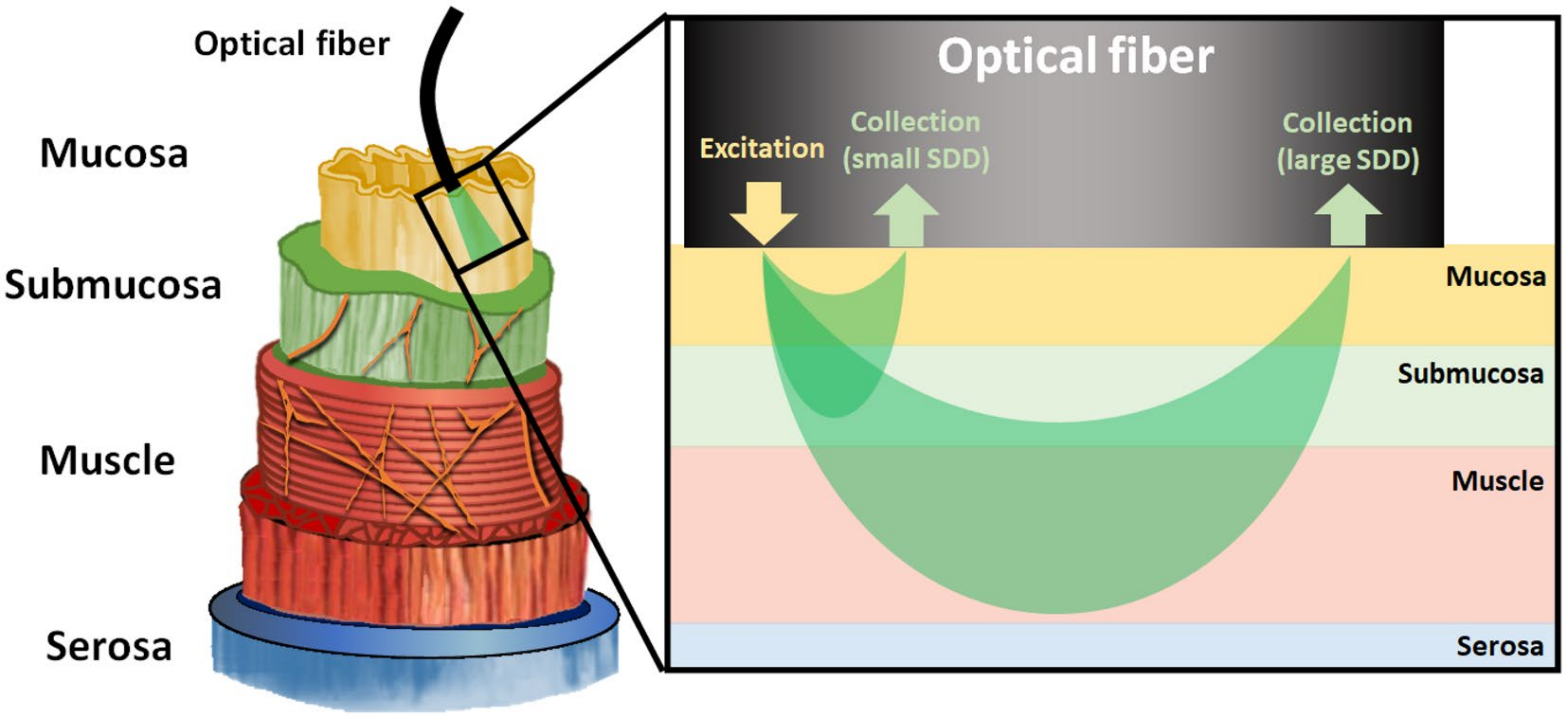
Tissue layers of the colon and rectum (left) and the light-tissue interaction volume interrogated by using the small SDD probe and the large SDD probe. The probed tissue depth increases with the SDD. Variations in the diffuse reflectance signal caused by carcinogenesis were evaluated based on the structural and biomolecular changes of the tissue layers investigated by each probe.

In normal mucosal tissues, the light that enters the colon from the luminal side is first absorbed by mucosal biomolecules (e.g. blood in mucosal capillaries) and scattered by collagen fibers and fibrils, epithelial cells, organelles, and cytoskeleton^80^. The diffuse-reflected light is composed of the scattering within the mucosal layer (Fig. 5) and the backscattered light at the boundary between mucosa and submucosa (refractive index mismatch). The diffuse-transmitted light enters the submucosal layer to be mostly absorbed by water and large submucosal vessels while scattered by the submucosal cellular and subcellular structures. Backscattering happens once more on the interface between the submucosa and muscle layers, which increases the percentage of diffuse-reflected light. In the muscle layer, large collagen bundles can scatter light forward. A small fraction of the light that was not absorbed by the muscle and serosa and backscattered within the muscle layer or in the interface with serosa is diffuse-reflected and may reach the light detector. The detected fraction of diffuse-reflected light of all tissue layers gives the DRS or elastic scattering spectroscopy signal. In tumor tissues, this fraction can be changed upon variation of optical properties resulting from biomolecular and structural associated to carcinogenesis^49^.

Most of the precancerous changes on the healthy colorectal mucosa originate in the epithelial tissue^80^. As the cancer progresses, changes due to angiogenesis as well as size and density of collagen fibers can be observed. Angiogenesis generates alterations in blood oxygen saturation and increases the density, volume and disorganization of the blood microvasculature. Cancerous tissues tend to have a high metabolism and an increase in blood volume in the tumor periphery, which can prevent the oxygen and nutrient supply to the center of larger tumors. This lack of oxygen and nutrient supply leads to necrosis and subsequent loss of central microvasculature in late-stage cancers^49^. Most of the tumors probed in this study are of advanced stages which may exhibit more pronounced structural and biomolecular changes. However, these changes are expected to progress over tumor stages and thus, the identification of parameters for optimized early-stage tumor detection requires feasibility studies including tumors of diverse stages such as the present study. Parameters to be optimized include the extended wavelength range and larger SDD explored in this study and discussed in more detail in “Wavelength selection and probed depth” section. In addition, DRS and optical spectroscopy are in the process of being introduced as a feasible tool for cancer detection in general, as already discussed in “Introduction” section. The use of DRS and hyperspectral imaging for CRC detection is discussed in the “Wavelength selection and probed depth”, “Comparison with DRS-related optical spectroscopy studies” and “Comparison with hyperspectral imaging studies” sections. Limitations due to the dataset based on tumors visible by naked eye are discussed in the “Limitations and validity of this study” section.

In terms of changes to be probed with optical techniques for early-stage cancer detection, Backman and Roy^80^ have shown that changes associated with carcinogenesis involve microvessel density in the mucosa (pericryptal network) and the superficial submucosa as well as structural changes on the mucosal crypts and stroma around 200–300 µm from the tissue surface (information from pictures of histology slides shown by the authors). Other suggested optically detectable alterations cited by the authors included nanostructural and microstructural changes in the chromatin structure, collagen fiber crosslinking and cytoskeleton.

### Wavelength selection and probed depth

**Figure 6:**
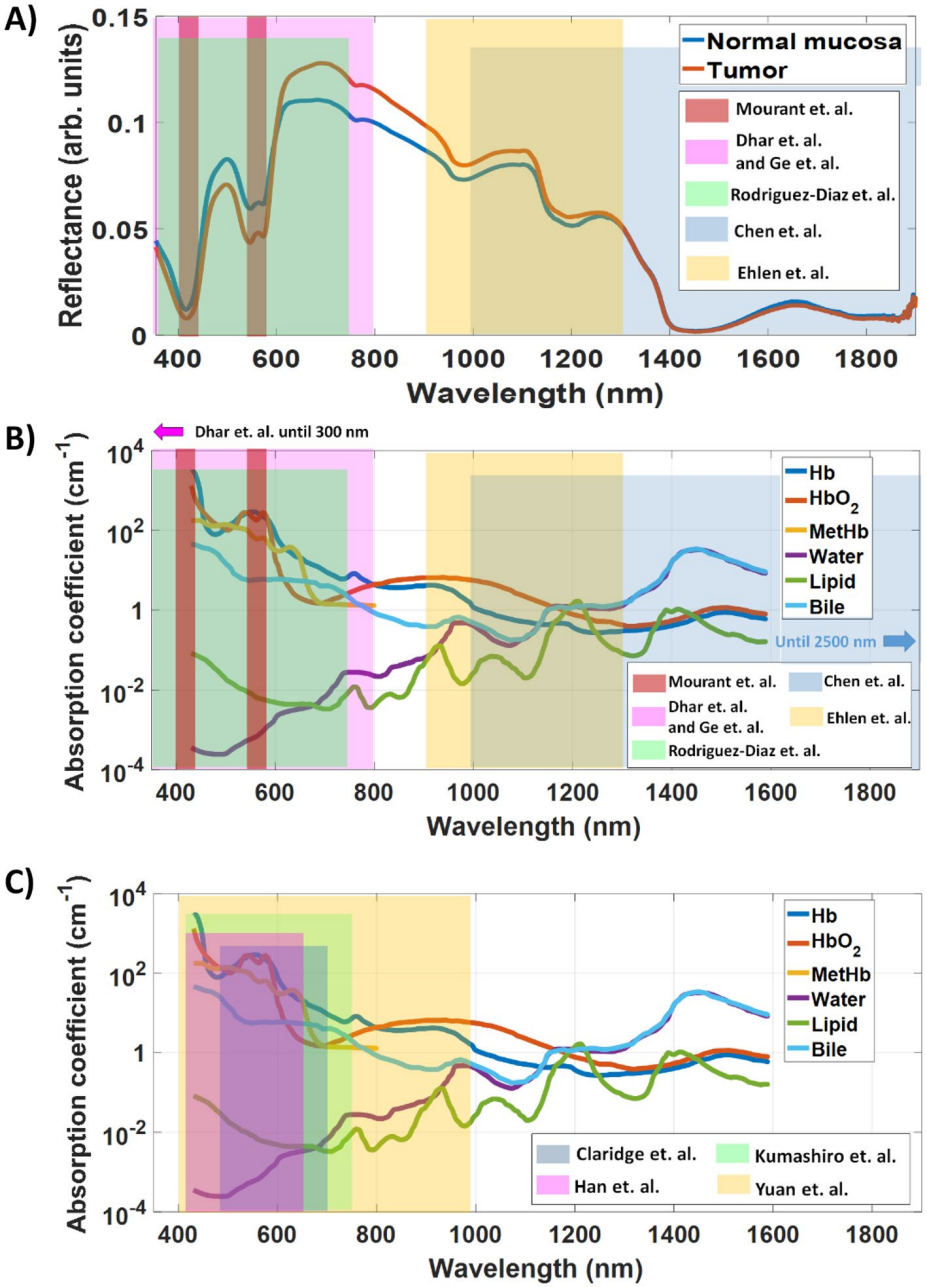
Wavelength ranges analyzed in previous DRS studies shown on the (**A**) mean spectra of normal mucosa and tumor (small SDD probe) collected in this study and (**B**) absorption spectra of endogenous tissue chromophores (biomolecules)^84^. (**C**) Absorption spectra of endogenous tissue chromophores showing the wavelength ranges investigated on previous hyperspectral imaging studies.

Previous DRS studies targeting cancer detection for colonoscopy guidance reported possible differentiation between luminal normal and premalignant or tumor tissue by using hemoglobin concentration and blood saturation information alone^39,40,81–83^. Similar considerations are made by studies evaluating hyperspectral imaging (HSI) for the same application^41,49,50^. Blood-related parameters are detected on the visible wavelength range, where the blood absorption is stronger (Fig. 6). Prior DRS and HSI studies focused on analysis using visible light, except by Chen et al.^45–47^, Ehlen et al.^48^ and Yuan et al.^42^. The typically targeted near-infrared wavelength region contains information about the water and lipid absorption. Longer wavelengths or shorter wavenumber also contain information about protein and carbohydrate content. The wavelength ranges used in prior studies are shown in Fig. 6.

In addition to the biochemical information of the wavelength ranges, the DRS signal contains tissue information on depths depending on the optical properties (absorption and scattering coefficients) and source-to-detector distance (SDD). Since the scattering coefficient is large on the visible wavelength range^53^, most of the light in this range is diffuse-reflected close to the source of light. Then, using small SDD probes allows efficient collection of visible emitted light and large SDD probes will collect visible light from deeper layers. However, the tissue scattering coefficient is relatively low in the near-infrared wavelength range, which allows the small SDD probes to collect the diffuse-reflected light from the deeper tissue layers, whereas large SDD probes collect light from even deeper layers. Therefore, the DRS probed depth is mainly determined by the SDD of the fiber optic probe.

Although DRS and HSI collect similar tissue information, there are major differences with respect to the probed depth and reflectance standardization. DRS is taken from point measurements in contact with the tissue. In this case, adjusting the probe SDD will control the depth to be probed. Yet, hyperspectral images are taken from a certain tissue area and, thus, each tissue point has a different distance from the detector^85^. This means the tissue reflectance is not standardized, as the standardization process requires every tissue point to be at a fixed distance from both source and detector. Since HSI works by illuminating a tissue area right below the light source and detecting the reflected intensity from the same area, the detected light comes primarily from the tissue surface^85^. Similarly, to the smaller SDD probes for DRS, the wavelength range (and optical properties) will have a strong effect on the probed tissue depth.

### Comparison with DRS-related optical spectroscopy studies

By using quadratic SVMs, we analyzed the performance of classification models using the wavelength range of previous studies. Since prior studies used only small SDD probes, we used the dataset of the 630-µm-SDD probe to build the classification models. As already shown in Fig. 6, 4 of these studies focused their analysis on features of the visible wavelength range and near-infrared (NIR) range until 800 nm, whereas 4 studies analyzed the wavelengths higher than 900 nm. In general, studies probing wavelengths lower than 800 nm exhibit lower classification performance for tissue differentiation compared to studies in the NIR range above 900 nm (Table 5). Yet, it is important to note that most of the studies including visible wavelengths were conducted in vivo and with a larger sample size compared to the NIR studies above 900 nm.

**Table 5:** Comparison of clinical data, instrument specifications and classification performance metrics across optical spectroscopy studies.

Authors	Tissue evaluation	Patients	Wavelength range (analysis)	Source-to-detector distance (SDD)	Tissues considered for analysis	Number of spectra analyzed	Classification	Sensitivity (%)	Specificity (%)	Accuracy (%)	AUC
Mourant et al.^39^	In vivo	15	Analyzed range: 400–440 nm and 540–580 nm collected range: 300–750 nm)	300–400 µm	21 normal mucosa, 31 colitis, inflammatory bowel disease, Crohn’s disease, 1 hyperplastic tissue, 6 adenomas and 1 adenocarcinoma	60 (1 spectrum per tissue site)	Non-cancer and cancer and tissues	100	98	–	–
Nogueira et al.^56^	Ex vivo	47	400–440 nm and 540–580 nm	630 µm	47 tumor and 47 normal tissues	1363 (728 normal mucosa and 635 cancer spectra)	Normal mucosa and cancer tissue	84.2 ± 2.9	86.3 ± 2.2	85.2 ± 2.6	0.917 ± 0.012
Dhar et al.^40^	In vivo	45	300–800 nm		138 colonic sites	483 (290 normal, 19 hyperplastic, 69 adenomatous polyps, 74 chronic colitis, and 31 colorectal cancer spectra)	Cancerous and normal tissue	80	86	–	–
							All pathological tissues and normal tissue	92	82	–	–
Ge et al.^43^	In vivo	153	350–800 nm	16 randomly placed fibers for tissue excitation and collection	107 non-neoplastic tissues (84 normal mucosa, 23 hyperplastic polyps) and 53 neoplastic tissues (44 adenomatous polyps, 9 adenocarcinomas)	244 (9 adenocarcinomas, 90 adenomatous polyps, 33 hyperplastic pol- yps, and 84 normal colon tissue spectra)	Neoplastic tissue from non-neoplastic tissue	79–91	74–91	–	–
							Adenomatous polyps from hyperplastic polyps	85–91	72–75	–	–
Nogueira et al.^56^	Ex vivo	47	350–800 nm	630 µm	47 tumor and 47 normal tissues	1363 (728 normal mucosa and 635 cancer spectra)	Normal mucosa and cancer tissue	87.4 ± 2.1	87.3 ± 2.7	87.4 ± 2.4	0.933 ± 0.009
Rodriguez-Diaz et al.^44^	In vivo	83	330–760 nm	250 µm	85 neoplastic tissue, 80 hyperplastic polyps and 53 polypoid colonic mucosa	218 spectra averaged from 1090 measurements (5 spectra per tissue)	Neoplastic and non-neoplastic tissues	91.5	92.2	91.9	-
Nogueira et al.^56^	Ex vivo	47	350–760 nm	630 µm	47 tumor and 47 normal tissues	1363 (728 normal mucosa and 635 cancer spectra)	Normal mucosa and cancer tissue	84.7 ± 2.4	87.0 ± 2.3	85.8 ± 2.4	0.922 ± 0.014
Chen et al.^45^	Ex vivo	20	1000–2500 nm (wavenumber interval from 4000 cm^-1^ to 10,000 cm^-1^)	Multiple (7.0 mm^2^ of sampling area)	78 cancer and 108 normal tissues	78 tumor spectra and 108 normal tissue spectra	Normal and cancer tissues	92.3	100		–
Chen et al.^46^	Ex vivo	–	1000–2500 nm	Multiple (7.0 mm^2^ of sampling area)	55 cancer and 55 normal tissue slices (stabilized in 4% formaldehyde)	110 (55 cancer and 55 normal tissue spectra)	Normal and cancer tissues	100	78.6	89.1	–
Chen et al.^47^	Ex vivo	–	1000–2500 nm	Multiple (7.0 mm^2^ of sampling area)	58 paraffin tissue sections	58	Normal and cancer tissues	92.8	86.7	–	–
Nogueira et al.^56^	Ex vivo	47	1000–1919 nm	630 µm	47 tumor and 47 normal tissues	1363 (728 normal mucosa and 635 cancer spectra)	Normal mucosa and cancer tissue	92.1 ± 1.2	92.3 ± 1.4	92.2 ± 1.3	0.95 ± 0.011
Ehlen et al.^48^	Ex vivo	10	Analyzed range: 900–1300 nm (collected range: 900–1720 nm)	400 µm + fiber cladding	10 tumor and 10 normal tissues	95 (50 tumor and 45 normal tissue spectra)	Normal mucosa and cancer tissue (DRS and FS)	98	93	96	–
							Normal mucosa and cancer tissue (only DRS)	93	68	82	
Nogueira et al.^56^	Ex vivo	47	900–1300 nm	630 µm	47 tumor and 47 normal tissues	1363 (728 normal mucosa and 635 cancer spectra)	Normal mucosa and cancer tissue	86.9 ± 3.5	88.0 ± 3.7	87.4 ± 3.6	0.921 ± 0.020

The dataset of this study (represented by the Nogueira et al.^56^ in the table) was evaluated in the wavelength ranges of previous studies and for comparison of classification results.

The classification performance metrics achieved in this study is comparable to most of the previous studies (Table 5), especially those probing NIR wavelengths above 900 nm and those including visible wavelengths with similar sample size. Non-comparable classification performance metrics are observed in studies by Mourant et al.^39^, who have investigated a substantially lower amount of spectra and patients, and Rodriguez-Diaz et al.^44^, who have used 250 µm of fiber SDD and probed different types of tissue compared to the present study. In terms of purely NIR studies, the group of Chen et al.^45–47^ showed the tissue discrimination achieved by using random forest, Adaboost and SVM classifiers. As expected, the closest classification performance compared to the present study was the SVM classifier. Still, Ehlen et al.^48^ found 6.1% higher sensitivity, 20% lower specificity and 5.4% lower accuracy compared to the present study, which may be originated by using 400-µm fiber SDD and substantially smaller sample size (number of patients and spectra).

The accuracy achieved in our study tends to be higher (i.e. due to increased sensitivity and/or specificity) compared to the in vivo DRS studies (Table 5). It is unclear whether higher accuracy is obtained due to differences in the tissue evaluation procedure (in vivo or ex vivo), number of patients, region where the study was conducted, types of interrogated tissue or machine learning model used in each study. At all cases, our classification model contains the highest number of investigated spectra and therefore, the largest representation over heterogeneity caused by probe contact pressure effects, probe positioning and other factors contributing to DRS signal variation during contact measurements.

### Comparison with hyperspectral imaging studies

In order to compare the performance metrics achieved by wavelength ranges of hyperspectral imaging (HSI) studies, we used quadratic SVM models build with the dataset of the 630-µm-SDD probe. The equivalent comparison comes from the information hyperspectral imaging gets from the superficial tissue, which is comparable to what is obtained by using the short SDD probe of our study. Most of the HSI studies have exploited the reflectance on the visible wavelength region and thus, based their tissue identification on blood absorption and relatively high superficial tissue scattering (Fig. 6, Table 6). Kumashiro et al.^50^ extended the wavelength range up to 750 nm and Yuan et al.^42^ extended the range further up to 1000 nm. Although the study of Yuan et al. investigated the water absorption signal at longer wavelengths, the study was limited by ex vivo samples of 4 patients.

**Table 6:** Comparison of clinical data, instrument specifications and classification performance metrics across hyperspectral imaging studies and this study.

Authors	Tissue evaluation	Patients	Wavelength range (analysis)	Normal and cancerous tissues considered for analysis	Number of images and spectra analyzed	Classification	Sensitivity (%)	Specificity (%)	Accuracy (%)	AUC
Claridge et al.^49^	Ex vivo	8	470–700 nm	7 adenocarcinomas, 2 adenomatous polyps, 1 adenoma, 1 tubulo-villous adenoma, 1 neoplastic polyp and 1 hyperplastic polyp and surrounding normal mucosa	–	Colorectal lesions and normal mucosa	85	78	–	–
Nogueira et al.^56^	Ex vivo	47	470–700 nm	47 tumor and 47 normal tissues	1363	Normal mucosa and cancer tissue	82.3 ± 2.2	86.0 ± 0.8	84.1 ± 1.6	0.900 ± 0.008
Kumashiro et al.^50^	Ex vivo	21	405–750 nm	40 tumor and 95 normal mucosa sites	135 (40 tumor and 95 normal mucosa images)	Normal mucosa and cancer tissue	72.5	82.1	–	–
						All pathological and normal tissues	75.0	55.8	–	–
Nogueira et al.^56^	Ex vivo	47	405–750 nm	47 tumor and 47 normal tissues	1363	Normal mucosa and cancer tissue	86.8 ± 0.5	89.0 ± 0.3	87.8 ± 0.4	0.927 ± 0.005
Han et al.^41^	In vivo	12	405–665 nm	21 colorectal tumors or adenomatous polyps and their surrounding normal mucosa	–	Normal mucosa and cancer tissue (all hyperspectral images)	96.9 ± 2.3	91.5 ± 4.1	94.5 ± 4.9	–
	In vivo	12	415–425 nm, 495–505 nm, 525–545 nm and the wavelengths 465 nm and 625 nm	21 colorectal tumors or adenomatous polyps and their surrounding normal mucosa	–	Normal mucosa and cancer tissue (selected wavelength bands)	94.7 ± 4.2	89.1 ± 3.2	92.9 ± 5.4	–
Nogueira et al.^56^	Ex vivo	47	405–665 nm	47 tumor and 47 normal tissues	1363	Normal mucosa and cancer tissue	85.6 ± 1.4	90.0 ± 0.7	87.7 ± 1.1	0.930 ± 0.010
Nogueira et al.^56^	Ex vivo	47	415–425 nm, 495–505 nm, 525–545 nm and the wavelengths 465 nm and 625 nm	47 tumor and 47 normal tissues	1363	Normal mucosa and cancer tissue	74.5 ± 0.4	83.0 ± 0.8	78.5 ± 0.6	0.863 ± 0.005
Yuan et al.^42^	Ex vivo	4	Analyzed: 405.235 nm, 406.42 nm, 408.794 nm, 414.736 nm, 422.48 nm, 468.76 nm, 559.489 nm, 577.506 nm, 594.342 nm, 957.948 nm, and 1,000.31 nm (collected: 400 nm and 1000 nm)	–	–	Cancerous and non-cancerous tissues	93.8	87.5	90.6	–
Nogueira et al.^56^	Ex vivo	47	400–1000 nm	47 tumor and 47 normal tissues	1363	Normal mucosa and cancer tissue	90.5 ± 1.2	92.0 ± 0.5	91.2 ± 0.9	0.963 ± 0.004
Nogueira et al.^56^	Ex vivo	47	405.235 nm, 406.42 nm, 408.794 nm, 414.736 nm, 422.48 nm, 468.76 nm, 559.489 nm, 577.506 nm, 594.342 nm, 957.948 nm, and 1,000.31 nm	47 tumor and 47 normal tissues	1363	Normal mucosa and cancer tissue	78.2 ± 0.7	86.0 ± 0.5	81.9 ± 0.6	0.903 ± 0.005

The dataset of this study (represented by the Nogueira et al.^56^ in the table) was evaluated in the wavelength ranges of previous studies and for comparison of classification results.

Studies including data on wavelengths between 400–470 nm showed higher classification performance compared to the study of Claridge et al.^49^, who used only wavelengths longer than 470 nm. This agrees with the results of our study and suggests that the blood content and oxygenation in superficial tissue layers highly contributes to the discrimination between mucosa and tumor tissues. In addition, even though Han et al.^41^ investigated a substantially narrower wavelength range than Yuan et al.^42^, they achieved higher classification performance in their in vivo study compared to an ex vivo study. This performance suggests that blood oxygenation changes in vivo, especially those probed between 405 and 665 nm, contribute significantly to the tissue differentiation. Still, further investigation is required to understand the origin of the contrast between normal and cancerous tissues by using HSI.

Previous HSI studies have investigated a significantly smaller patient population compared to DRS studies. However, the classification performance metrics obtained in this study as well as their changes due to wavelength ranges were comparable when considering similar specifications with respect to the probe depth investigated by either imaging from a distance or using lower SDD fiber probes. Compared to the performance metrics that could be achieved in this study by using the wavelength range between 405 and 665 nm, Han et al.^41^ obtained significantly higher metrics on their in vivo study by using support vector machines. Conversely, the ex vivo tissue classification performed on a wider wavelength range (from 405 to 750 nm) by Kumashiro et al.^50^ led to much lower performance metrics compared to our study.

Current hyperspectral imaging studies are limited by the number of patients, and lack of standardization on imaged angles, distance from the tissue and SDDs for each pixel. Therefore, comparison with performance metrics achieved by point spectroscopy (which standardizes measurement parameters and has proven to be robust among a large number of patients) still requires further investigation to address those limitations. On the other hand, HSI has the potential to cause a significant decrease in measurement times by acquiring data of multiple points at once. In order to keep the differentiation achieved by previous spectroscopic studies, HSI has to be used at optimum wavelengths for application-specific tissue identification.

### Limitations and validity of this study

Our paper has a number of limitations. First, this study does not include non-cancer pathology, which will be investigated in future research to incorporate data for improved tissue identification and possibly accurate sampling of areas to be biopsied. The measurements of this study have been performed in tumors of diverse stages with predominance of advanced stage tumors (Table 1). The tumor sites to be measured were identified by a combination of palpation and naked eye determination by experienced surgeons who demarcated the tumor region. However, this determination does not decrease the validity of our dataset and robust classification model, which is based on a larger dataset compared to previous studies (Tables 5, 6) and comprises variations on DRS spectra due to parameters discussed in this section. With this in mind, tissue alterations occurring in early-stage cancer (e.g. changes on the tissue microstructure due to cell proliferation) are expected to be the same as advanced cancer but with lower magnitude. Our feasibility study shows that these alterations are detectable and improved detection is achieved by larger SDD probes as well as extended wavelength ranges into the near-infrared.

Since the tissue blood oxygenation may change in ex vivo tissues during our measurements, we evaluated its changes over time in a pilot observation of the DRS signal in 3 patients and found no significant variations during the first 15 min of our data collection (measurements taken every 5 min and data not shown). Even though tissue oxygenation may be different between our research and prior in vivo studies due to collection of DRS measurements of ex vivo tissues, the trend observed in our data matches with that found in previous studies in general. Variations due to tissue blood oxygenation are expected to be similar to what was obtained by Baltussen et al.^9^, who reported that blood content and StO_2_ increased in the measured ex vivo within 1 h after resection in relation to in vivo tissues. Other factors changing ex vivo spectra include tissue dehydration, which was reduced as much as possible in this study by maintaining the tissue moisture with a wet wipe. However, it is important to remember that Baltussen et al.^9^ used a 1290-μm SDD probe and investigated fat, tumor, and healthy colorectal wall tissues.

In terms of variations observed in every technique requiring contact measurements, probe contact pressure and sufficient optical contact may be challenging to accomplish during in vivo endoscopy. If we consider that the large SDD probe collects data from a larger tissue volume and pressure is applied more uniformly during measurements, a reduced sensitivity to optical contact and probe positioning is expected. In this case, higher repeatability of measurements with the large SDD may be associated with the higher classification performance with the large SDD probe. Yet, the larger probed volume means the probe contact pressure may lead to variations due to the thickness of tissue layers. The variations commented above were previously reported^62^ and need to be further investigated in order to apply automated corrections to DRS spectra. Still, the measurements performed in this study incorporate variations due to probe contact pressure and temperature decrease in ex vivo tissues and a dataset substantially larger (1363 spectra for the small SDD probe and 1526 for the large SDD probe) than prior studies was generated in order to build a robust classification model.

Finally, other types of classification methods (e.g. deep neural networks) can be used to build classification models that can easily be run in the real-time. In fact, we tested other types of classification methods which resulted in sensitivities and specificities higher than 85% (data not shown). High performance metrics independent on the classification method used means our data clearly shows the discrimination between mucosa and cancer tissues. Therefore, we believe that future in vivo studies advancing research presented here should consider using larger SDD probes and extended wavelength ranges in the near-infrared for differentiation between mucosa and cancer tissues. As a factor for clinical translation, the incorporation of 2.5-mm-SDD probes is feasible, as the size of colonoscope channels range from 2.8 to 4.2 mm^86,87^.

## Conclusions

In this study, we evaluated the usefulness of the extended wavelength range for improving CRC detection using DRS and compared results achieved in this study with previous research. By using probing the superficial tissue, we obtained (93.5 ± 2.4)% sensitivity, (94.0 ± 1.7)% specificity and 0.971 ± 0.014 AUROC, whereas (96.1 ± 1.8)% sensitivity, (95.7 ± 0.6)% specificity and 0.987 ± 0.005 AUROC was achieved by sampling deeper tissue layers. To the best of our knowledge, this is the first DRS study to investigate the potential of probing deeper tissue layers using larger SDD probes for CRC detection in the luminal wall. Our study was conducted in ex vivo tissues, while it is straight forward to extend this methodology to an in vivo examination during endoscopy. Future studies employing diffuse reflectance spectroscopy, elastic scattering spectroscopy, near-infrared spectroscopy, hyperspectral imaging and spatial frequency domain imaging can exploit enhanced tumor detection due to the use of large SDD probes and the broadband wavelength range illustrated in this study. In a practical perspective, this study could potentially be used to develop a probe for CRC detection during colonoscopy. Real-time tissue classification is enabled by automated SVM model coupled with a DRS instrument capable of displaying the result of a single reading in about 2–3 s. By integrating this capability into a flexible fiberoptic probe which could be passed down a scope working channel, optical spectroscopy can obviate the need for multiple biopsies or polypectomies of normal mucosa as well as identify more subtle mucosal abnormalities such as sessile serrated polyps for example, which may be difficult to recognize during colonoscopy.
